# Microbiota Modulates Behavior and Protein Kinase C mediated cAMP response element-binding protein Signaling

**DOI:** 10.1038/srep29998

**Published:** 2016-07-21

**Authors:** Li Zeng, Benhua Zeng, Haiyang Wang, Bo Li, Ran Huo, Peng Zheng, Xiaotong Zhang, Xiangyu Du, Meiling Liu, Zheng Fang, Xuejiao Xu, Chanjuan Zhou, Jianjun Chen, Wenxia Li, Jing Guo, Hong Wei, Peng Xie

**Affiliations:** 1 Department of Neurology, The First Affiliated Hospital of Chongqing Medical University, Chongqing, China; 2 Institute of Neuroscience and the Collaborative Innovation Center for Brain Science, Chongqing Medical University, Chongqing, China; 3 Chongqing Key Laboratory of Neurobiology, Chongqing, China; 4 Department of Laboratory Animal Science, College of Basic Medical Sciences, Third Military Medical University, Chongqing, China; 5 Key Laboratory of Clinical Laboratory Diagnostics (Ministry of Education), Department of Laboratory Medicine, Chongqing Medical University, Chongqing, China; 6 Chongqing Cancer Hospital & Institute & Cancer Center, Chongqing, China; 7 Department of Neurology, Yongchuan Hospital, Chongqing Medical University, Chongqing, China; 8 South Australian Health and Medical Research Institute, Mind and Brain Theme, and Flinders University, Adelaide, SA, Australia

## Abstract

Evolutionary pressure drives gut microbiota–host coevolution and results in complex interactions between gut microbiota and neural development; however, the molecular mechanisms by which the microbiota governs host behavior remain obscure. Here, we report that colonization early in life is crucial for the microbiota to modulate brain development and behavior; later colonization or deletion of microbiota cannot completely reverse the behaviors. Microarray analysis revealed an association between absence of gut microbiota and expression in cAMP responding element-binding protein (CREB) regulated genes in the hippocampus. The absence of gut microbiota from birth was shown to be associated with decreased CREB expression, followed by decreases of protein kinase C beta (PRKCB) and AMPA receptors expression, and an increase of phosphorylation CREB (pCREB) expression. Microbiota colonization in adolescence restored CREB and pCREB expression, but did not alter PRKCB and AMPARs expression. The removal of the gut microbiota from SPF mice using antibiotics only reduced pCREB expression. These findings suggest that (i) colonization of the gut microbiota early in life might facilitate neurodevelopment via PKC–CREB signaling and (ii) although GF mice and ABX mice display reduced anxiety-related behaviors, the molecular mechanisms behind this might differ.

In addition to their role as symbionts, the trillions of microbes that inhabit our bodies are important for the internal environment. These microbes are most abundant in the gut, reaching 1 × 10^13^ to 1 × 10^14^ organisms per gram, and containing 100 times more genes than the host. The gut microbiota assembles during the first 2–3 years of postnatal life and can be modified by host genes, diet, and antibiotics. There is growing evidence that a change in the gut microbiota community can contribute significantly to human biology and development, as well as resulting in diseases, including obesity-related diseases, immune disease, gastrointestinal, and liver diseases^1^,^2^,^3^. However, recent research in mice has indicated that changes in the gut microbiota early in life are particularly influential, raising the possibility that it can play a role in an organism’s development and function elsewhere in the body^4^,^5^.

The brain is the organ that is most susceptible to both internal and external environmental factors in adolescence and early adulthood. During this period, the neuronal architecture and function undergo rapid modulation to cope with environmental challenges, resulting in cognitive and behavioral changes^6^. The hippocampus is closely associated with memory, cognition, and emotion. Emerging evidence indicates that the gut microbiota can modulate the hippocampus in a way that results in psychiatric disorders, such as major depression and autism^7^,^8^,^9^,^10^. The composition of the gut microbiota in these patients differs from that in the general population^10^,^11^, and in the mice model reintroduction of commensal bacteria has been shown to improve autism-related behavioral abnormalities^8^,^12^,^13^.

All of these studies used germ-free (GF) model mice to mimic the features of diseases, in which the gut microbiota is absent at the postnatal stage and the effects of the gut microbiota on neurodevelopment can be directly assessed. Another model lacking gut microbiota can be produced by removing the gut microbiota from specific-pathogen-free (SPF) mice using antibiotics (ABX)^14^,^15^. A previous study demonstrated that both GF mice and ABX mice have anomalous gene expression in the hippocampus, which was associated with more exploratory and less anxiety-like behaviors and exaggerated stress responses, as regulated by the hypothalamic–pituitary–adrenal (HPA) axis^16^,^17^,^18^. Moreover, the enhanced activity of the HPA axis in GF mice could be partially reversed by subjecting them to colonization (hereafter referred to as CGF mice) using fecal matter from SPF mice, and the effect dependent on the time since colonization^19^. Multiple pathways may be involved in the microbiota–gut–brain axis, such as the endocrine, immune, and neural pathways (vagus and enteric nervous system pathways)^20^. However, it remains unclear which pathway plays the most important role in this axis. To obtain a more comprehensive picture of microbiota–brain axis, we transplanted the gut microbiota of SPF to GF mice and removed the gut microbiota from SPF mice to investigate the behavioral and molecular of hippocampus changed by gut microbiota.

## Materials and Methods

### Animals

SPF and GF Kunming (KM) male mice (aged 6–8 weeks) were provided by the Department of Laboratory Animal Science of the Third Military Medical University (Chongqing, China). GF mice were kept in flexible film gnotobiotic isolators until the beginning of the behavioral tests. The germ-free status of the mice was verified by testing the feces and skin in accordance with Chinese Laboratory Animal-Microbiological Standards and Monitoring (GB 14922.2-2011) and PCR analysis using a universal primer for the V3 region of the bacterial 16s rRNA gene, as described previously^21^,^22^. All animals were group-housed and fed the same autoclaved chow and water ad libitum under a 12-h light–dark cycle (lights on at 7:30 a.m.), with a constant temperature of 21–22 °C and humidity of 55 ± 5%. The experiments followed the National Institutes of Health Guide for the Care and Use of Laboratory Animals (NIH Publication No. 80-23), revised in 1996, and were approved by the Ethics Committee of Chongqing Medical University.

#### Microbiota colonization

For the colonization experiments, a group of 4–5-week-old GF mice (n = 21) were placed in cages with bedding material preconditioned by exposure to SPF mice for 3 weeks, a protocol that was previously demonstrated to be effective at restoring a normal microbiota^23^.

#### Antibiotic treatment

A group of 4-week-old SPF mice (n = 15) were provided with drinking water containing 1 mg ml^−1^ cefoxitin (Haiyao Ltd., Hainan, China), 1 mg ml^−1^ gentamicin (Vita Ltd., Shanghai, China), 1 mg ml^−1^ metronidazole (Sangon Ltd., Shanghai, China), and 1 mg ml^−1^ vancomycin (Sangon Ltd., Shanghai, China), for 4 weeks ad libitum, as previously described^15^,^18^. Antibiotics were renewed every other day. Control mice (n = 15) received no antibiotics. All animals were group-housed and their fluid consumption was measured individually to calculate the amount of antibiotic received by each mouse. Body weight and food consumption of each mouse at the end of each week were used to assess the effects of the antibiotic.

### Behavioral procedures

On the test day, the GF mice (n = 31) and the CGF mice (n = 21) were removed from the isolators, transferred to the testing room with SPF mice (n = 32), and acclimated to the conditions there for at least 1 h. After each individual test session, the apparatus was thoroughly cleaned with 70% alcohol to eliminate the odor and traces of the previously tested mouse, as reported previously^24^.

#### Open field test (OFT). The OFT was conducted based on previously described procedures

Mice were placed individually in a corner of the open field arena (45 × 45 × 45 cm) and allowed to explore freely for 6 min. The testing arena was brightly lit. Activity was recorded using a video tracking system and analyzed using SMART 2.5 software (Panlab, Barcelona, Spain). The spontaneous activities performed by the mice were measured in the last 5 min^11^,^25^,^26^.

#### Forced swim test (FST)

The FST was conducted based on a previously described procedure^27^. In the first test, each animal was immersed in water for 15 min and tested with a second swim 24 h later. The mouse was placed in a Plexiglas cylinder (30 cm height, 15 cm diameter) filled with water at 25 °C to a height of 18 cm. After the test, the animal was dried under a lamp for 30 min. Immobility was defined as passive floating, excluding the minimal movements required for the mouse to keep its head above the water. Struggling was defined as vigorous movements with the forepaws breaking the water. All FST experiments were recorded by a video tracking system and analyzed using SMART 2.5 software by surface change rate between sequential images of animal shape. The activity was defined as immobility or struggling if the pixel change rate was lower than 4 cm s^−1^ or higher than 8 cm s^−1^, respectively. The duration of immobility or struggling for the last 5 min of the 6-min test period was measured.

### Microarray analysis

Transcriptional profiles of the hippocampus were assessed using Mouse LncRNA Array v2.0 (8 × 60 K, Arraystar). Briefly, hippocampal tissues from three mice were combined to obtain a pooled RNA sample, and all experiments in the GF group, the SPF group, and the CGF group were performed in triplicate using distinct samples. Total RNA from each sample was quantified using the NanoDrop ND-1000 and the RNA integrity was assessed using standard denaturing agarose gel electrophoresis. For microarray analysis, the Agilent Array platform was employed. Sample preparation and microarray hybridization were performed based on the manufacturer’s standard protocols with minor modifications. Briefly, mRNA was purified from total RNA after the removal of rRNA (mRNA-ONLY™ Eukaryotic mRNA Isolation Kit, Epicentre). Then, each sample was amplified and transcribed into fluorescent cRNA along the entire length of the transcript without 3′ bias, using a random priming method. The labeled cRNAs were hybridized onto the Mouse LncRNA Array v2.0 (8 × 60 K, Arraystar). After washing the slides, the arrays were scanned by the Agilent Scanner G2505C. Agilent Feature Extraction software (version 11.0.1.1) was used to analyze the acquired array images. Quantile normalization and subsequent data processing were performed using the GeneSpring GX v11.5.1 software package (Agilent Technologies). After quantile normalization of the raw data, mRNAs for which at least three out of nine samples had flags in Present or Marginal (“All Targets Value”) were chosen for further data analysis. Differentially expressed mRNAs with statistical significance between two groups were identified through Volcano Plot filtering.

### Bioinformatic analysis

Bioinformatic analysis was performed with Ingenuity Pathway Analysis (IPA) software (Ingenuity Systems, Redwood City, CA, USA; www.ingenuity.com). A comparative analysis was carried out to identify the most significant biological processes and canonical pathway in the dataset (p < 0.05 calculated using two-tailed Fisher’s exact test).

### Quantitative real-time PCR

RNA was also extracted from flash-frozen hippocampal tissue using the Trizol extraction protocol (Invitrogen, CA, USA), as mentioned previously, and reverse-transcribed into cDNA using a PrimeScript RT Reagent Kit (Takara, Dalian, China). qRT-PCR was performed on the LightCycler 96 system (Roche, Germany) using FastStart Essential DNA Green Master (Roche), in accordance with the manufacturer’s recommendations. Validated primer sets were obtained from Primerbank (Harvard) and commercially synthesized by Sangon Biotech (Shanghai, China). Target mRNA values were normalized using β-actin mRNA and data are expressed relative to the normalized values of corresponding controls.

### Western blotting

Western blotting was conducted based on previously described procedures with minor modifications^28^. The hippocampus was dissected from mice, immediately frozen on dry ice, and stored at −80 °C. Samples were thawed on ice as needed and homogenized in ice-cold Syn-PER synaptic Protein Extraction Reagent (Thermo Fisher) containing 1× protease inhibitor cocktail and 1× phosphatase inhibitors (Roche). The homogenate was centrifuged at 1,200 g for 10 min at 4 °C. The supernatant was then transferred to a new tube and centrifuged at 15,000 g for 20 min at 4 °C. After centrifugation, the supernatant was removed and the pellet was resuspended and sonicated in Syn-PER reagent. Protein concentration was determined by the BCA protein assay. The samples were resolved by SDS-PAGE and transferred onto polyvinylidine fluoride (PVDF) membranes. The PVDF membranes were blocked with 5% non-fat milk in Tris-buffered saline–0.05% Tween-20 (TBST) for 1 h at room temperature and incubated with primary antibodies overnight at 4 °C. Primary antibodies for the following molecules were used: cyclic AMP-responsive element-binding protein (CREB; 1:1,000; Cell Signaling Technology), phospho-CREB (Ser133) (1:1,000; Cell Signaling Technology), and β-actin (1:10,000; Ruiying Biological). The membrane was then washed three times for 10 min in TBST and incubated with horseradish peroxidase-conjugated anti-rabbit IgG (1:5,000 to 1:10,000; Bio-Rad) for 2.5 h. After three washes with TBST, the membrane film was developed using the enhanced chemiluminescence reagent (Millipore). The chemiluminescence signal was imaged using film (Fujifilm, Tokyo, Japan). The intensities of the stained lanes and immunoblots were quantified using Quantity One software (Bio-Rad).

### Statistical analysis

Statistical analysis was performed using GraphPad Prism 6. Differences between groups were assessed using one-way ANOVA with Fisher’s least significant difference test. Significant differences identified using the above tests are indicated in the figures as follows: *p < 0.05, **p < 0.01, and ***p < 0.001. Notable near-significant differences (p < 0.1) are also indicated in the figures. Data are reported as the mean ± SEM.

## Results

### Changes in gut microbiota alter behaviors

**Figure 1:**
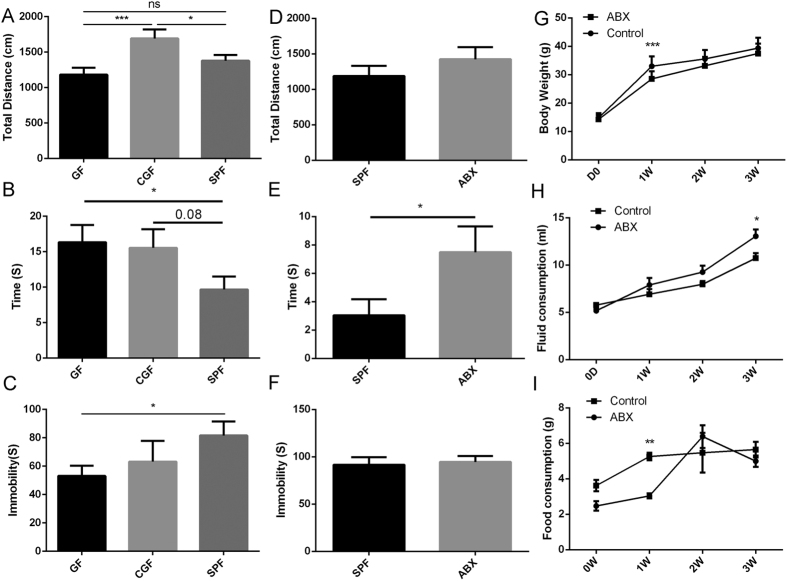
Alteration of the gut microbiota changed host behaviors. (**A–C**) Total distance traveled (**A**) and time spent in the center (**B**) in the open field test (OFT) and immobility time (**C**) in the forced swim test (FST) for GF mice (n = 31), CGF mice (n = 21), and SPF mice (n = 32) were assessed at the end of the experiment. (**D–I**) During antibiotic treatment, total distance traveled (**D**) and time spent in the center (**E**) in the OFT, immobility time (**F**) in the FST, body weight (**G**), fluid consumption (**H**) and food consumption (**I**) for ABX mice (n = 15) and SPF mice (n = 15) were assessed every week for 3 weeks. Data represent the mean ± S.E.M. *p < 0.05, **p < 0.01, ***p < 0.001; ns, not significant.

Initially, to determine whether the host microbiota can alter host behaviors, we subjected adult GF and SPF mice to a battery of tests for anxiety-related and passive behaviors. Consistent with previous research, GF mice explored the center of the open field more (Fig. 1A,B). Moreover, we found that GF mice were immobile for less time than SPF mice in the FST (Fig. 1C). However, CGF mice exhibited significant increases in their motor activity and time spent in the center of the OFT compared with SPF mice (Fig. 1A,B), as well as increased motor activity compared with GF mice (Fig. 1B). Their exploration of the center and antidepressant behaviors were similar to those of GF mice (Fig. 1B,C).

The removal of the gut microbiota from SPF mice on 4-week-old using antibiotics for 3 weeks did not produce significant changes in the immobility time of the FST and total distance traveled in the OFT (Fig. 1D,F), but did increase the central time spent in the OFT (Fig. 1E). To illustrate the effects of the antibiotic on the results of the behavioral tests, we measure the fluid and food consumption and weight gain in the two groups. We found that fluid consumption did not differ significantly between the two groups at the ends of the first two weeks; however, at the end of the third week, the fluid consumption of the ABX group was greater than that of the control group (Fig. 1H). Moreover, the ABX group gained less weight than the control group in the first week, but there were no significant differences in body weight between the two groups at the ends of the last two weeks (Fig. 1G). The ABX group consumed less food than the control group in the first week, which might explain why that group gained less weight in this period. However, there were no significant differences in food consumption between the two groups in the last two weeks (Fig. 1I).

**Figure 2:**
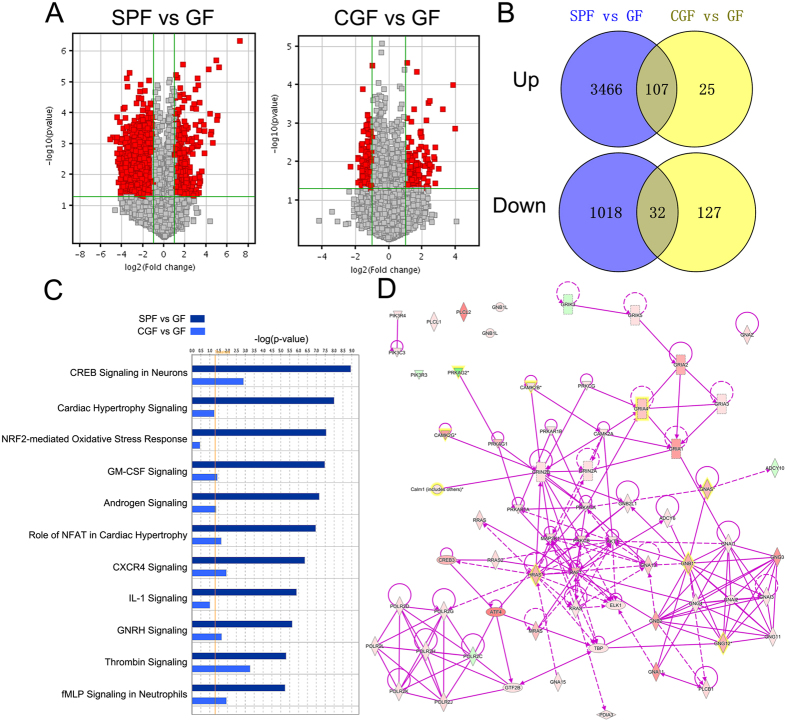
Transcript analysis of microbiota–brain axis. (**A**) A volcano plot presents the expression of signature genes of the hippocampus in SPF mice and CGF mice compared with GF mice. (**B**) A Venn diagram indicating the number of genes differentially expressed in SPF mice and CGF mice compared with GF mice. (**C**) Ingenuity Pathway Analysis and Canonical Pathway Analysis of the expression of signature genes from three datasets in the hippocampus related to gut microbiota.

To investigate the transcriptional alterations relevant to gut microbiota, we profiled the hippocampal samples collected from GF and SPF mice. A volcano plot of the microarray data shows the transcripts that exhibited differential expression between SPF mice and GF mice (Fig. 2A). The microarray study identified 1068 upregulated mRNAs and 3591 downregulated mRNAs in the hippocampus of GF mice (≥2.0-fold) (Fig. 2B). However, whether differential gene expression can be reversed by colonization of the gut by microbiota has remained unclear. We thus compared the mRNA differential expression levels in the hippocampus between GF mice and CGF mice (Fig. 2A). Among these mRNAs, 107 upregulated and 32 downregulated mRNAs were significantly restored (Fig. 2B). The IPA database was used to identify the functional connections among these genes (Fig. 2D). Canonical Pathway Analysis was also used to display the most significantly altered genes relevant to the gut microbiota (Fig. 2C).

### Commensal microbiota influences CREB signaling in the hippocampus

**Figure 3:**
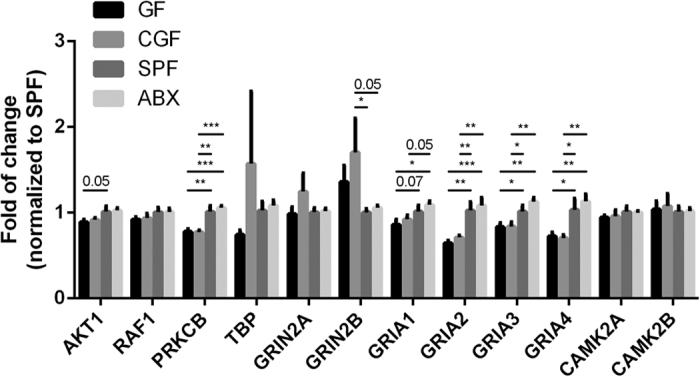
Validation of CREB signaling change in the hippocampus. Real-time PCR validation of selected differentially expressed genes involved in CREB signaling among GF mice (n = 6), CGF mice (n = 6), SPF mice (n = 6), and ABX mice (n = 6). Data represent the mean ± S.E.M. *p < 0.05, **p < 0.01, ***p < 0.001.

In our analysis, CREB signaling was found to be one of the most highly dysregulated signaling pathways related to gut microbiota; it regulates the expression of genes involved in neuroplasticity, cell survival, cognition, and behavior. In an attempt to determine whether CREB signaling is regulated by the gut microbiota, we investigated 13 genes of the modulated genes involve CREB signaling. We confirmed that the expression of four genes (PRKCB, GluR2, GluR3, GluR4) was significantly decreased in GF mice compared with that in SPF mice, and that microbiota colonization in GF mice could not restore their expression. However, the expression of two genes (Akt1, GluR1) was decreased in GF mice compared with that in SPF mice, but could be partially restored by microbiota colonization. NMDAR2B showed no significant difference in expression between GF mice and SPF mice, but after colonization of the gut microbiota to GF mice, its expression increased in CGF mice compared with that in SPF mice. Antibiotic treatment could not change the expression of these genes compared with that in SPF mice (Fig. 3A).

**Figure 4:**
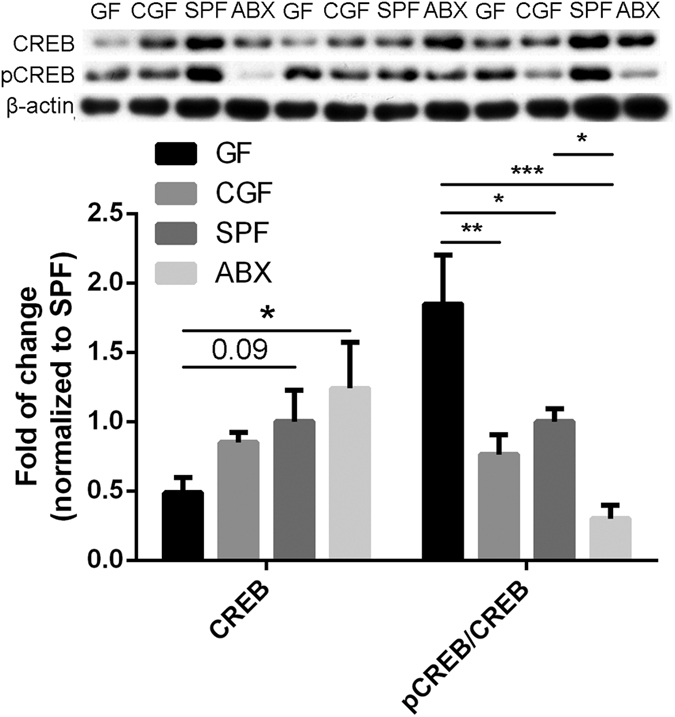
Effect of the gut microbiota on CREB expression. Immunoblotting analysis of CREB and phospho-CREB expression in the hippocampus of GF mice (n = 3), CGF mice (n = 3), SPF mice (n = 3), and ABX mice (n = 3). Total protein was extracted and subjected to SDS-PAGE, followed by Western blot analysis, all experimental under the same conditions. β-actin was used as an internal control. The upper panel shows a representative Western blot result. The lower panel illustrated the fold difference of integrated absorbance of protein after normalization with β-actin. Data represent the mean ± S.E.M. *p < 0.05, **p < 0.01, ***p < 0.001.

Moreover, we used immunoblotting to clarify the CREB and the CREB protein expression in the hippocampus in our models (Fig. 4). Western blot analysis revealed that the gut microbiota caused the level of CREB to increase and that of pCREB to decrease. Colonization slightly increased CREB and markedly decreased pCREB, but antibiotic treatment could not restore this. ABX mice displayed increased CREB expression compared with GF mice and decreased pCREB expression compared with the other three groups.

## Discussion

The regulation of emotion and behavior is complex and heterogeneous, which has been considered to be the outcome of gene–environment interactions^29^. Gut microbiota as the largest ecosystem in the body affects myriad physiological features, including neuronal function and behaviors. The GF model provides a direct way of characterizing the neuronal network and behaviors regulated by the gut microbiome. GF animals have been shown to exhibit altered anxiety-like and social behaviors^11^,^16^,^30^,^31^,^32^,^33^,^34^. However, there is controversy about how the gut microbiota affects anxiety behaviors. Previous studies demonstrated reduced anxiety in GF NMRI and Swiss mice, while GF F344 rats and BALB/c mice showed increased anxiety compared with SPF mice^16^,^31^,^32^,^34^,^35^. Several factors have been proposed to explain the reported differences, including hyperactivity of the HPA axis and change in the composition of neurochemical and inflammatory factors in GF mice. However, some of the aforementioned studies did not explore the relationship between the change of gut microbiota and behavioral and molecular expression.

Our screening showed that several classes of gene may be involved in the microbiota–brain axis, including protein kinases, receptors, transcription factors, and ion channels. The bioinformatic analysis revealed that the CREB signaling pathway is most significantly associated with the microbiota–brain axis. CREB belongs to the leucine zipper transcription factors family, which is expressed ubiquitously in tissues and cells. Homeostasis of CREB signaling is important for synaptic plasticity and cerebral senior function. CREB signaling disturbance in the hippocampus of GF mice was first mentioned by Diaz Heijtz *et al*.; subsequently, Stilling *et al*. proved that CREB target genes were upregulated in the amygdala of GF mice, but colonization by conventional microbiota completely failed to restore their expression to normal levels^34^,^36^. Our immunoblotting results indicate that CREB activation in the hippocampus was significantly increased in response to an absence of the gut microbiota and could be restored by microbiota colonization. Removal of the gut microbiota by antibiotics in adolescence decreases CREB activation, which impairs long-term potentiation in the hippocampus, leading to the poor encoding of memories. These results indicate that, in GF mice and ABX mice, different molecular mechanisms are involved in reducing anxiety-related behaviors.

A number of pathways are involved in the activation of CREB, including the cAMP pathway, the protein kinase pathway, and the PI3K/Akt pathway. In our study, PRKCB decreased in GF mice, and the recolonization or removal of gut microbiota in adolescence could not reverse its expression. There was no significant difference in CaMK2A, CaMK2B, and TBP among the four groups, but the level of Akt slightly decreased in GF mice. In general, the PKC signaling pathway is one of the most commonly used pathways in neurons; it activates and phosphorylates CREB, so suppression of the PKC pathway would inhibit CREB expression and activation^37^. Although both Akt and PKC are known to phosphorylate CREB, increasing pCREB in GF mice might be a way to compensate for a low level of total CREB expression following the suppression of PKC.

Glutamatergic synapses are the most abundant excitatory transmission structure in the central nervous system and are regulated by CREB signaling^38^,^39^. A previous study demonstrated that the loss of CREB function decreased the expression of glutamatergic receptors^39^. Consistent with this study, our results show that, upon the downregulation of AMPARs following decreased CREB expression in GF mice, the restoration or removal of microbiota in adolescence could not change the gene expression. The alteration of AMPARs represents a common underlying pathology in learning and memory, and even in mood disorders. For instance, mice lacking GluR1 exhibit impaired spatial working memory and those lacking GluR2 exhibit reduced synaptic transmission and major motor abnormalities^40^. These results demonstrate that colonization of the gut by microbiota early in life is crucial for brain development.

There are several limitations to this study. First, antibiotic treatment cannot completely remove the gut microbiota^18^, so it cannot be ruled out that some of the residual microbiota increased anxiety-related behaviors. Moreover, long-term administration of a high dose of antibiotics might produce chronic stress. Second, more samples should have been obtained to verify the function of PKC–CREB signaling in the gut–microbiota–brain axis. Third, CREB is phosphorylated by multiple signaling pathways, so further work is necessary to clarify whether other pathways participate in the activation of CREB in GF mice.

In summary, the results of this study suggest that colonization of the gut by microbiota after birth is critical for brain development and behavioral regulation. The discovery of the molecular mechanism behind the microbiota–brain axis provides insight into the coevolution of host–microbiota interactions brought about by evolutionary pressure.

## Additional Information

**How to cite this article**: Zeng, L. *et al*. Microbiota Modulates Behavior and Protein Kinase C mediated cAMP response element-binding protein Signaling. *Sci. Rep.* **6**, 29998; doi: 10.1038/srep29998 (2016).
